# Evaluation of deliverable artificial intelligence-based automated volumetric arc radiation therapy planning for whole pelvic radiation in gynecologic cancer

**DOI:** 10.1038/s41598-025-99717-y

**Published:** 2025-04-30

**Authors:** Yushan Xiao, Shohei Tanaka, Noriyuki Kadoya, Kiyokazu Sato, Yuto Kimura, Rei Umezawa, Yoshiyuki Katsuta, Kazuhiro Arai, Haruna Takahashi, Taichi Hoshino, Keiichi Jingu

**Affiliations:** 1 Department of Radiation Oncology, Tohoku University Graduate School of Medicine, 1-1 Seiryo-machi, Aoba-ku, Sendai, 980-8574 Japan; 2 Radiation Technology, Tohoku University Hospital, Sendai, Japan; 3 Radiation Oncology Center, Ofuna Chuo Hospital, Kamakura, Japan

**Keywords:** Deep learning, Whole pelvic radiation therapy, Deep learning based deliverable plan, Gynecologic cancer dose prediction, Radiotherapy, Cancer, Oncology, Cancer

## Abstract

This study aimed to develop a deep learning (DL)-based deliverable whole pelvic volumetric arc radiation therapy (VMAT) for patients with gynecologic cancer using a prototype DL-based automated planning support system, named RatoGuide, to evaluate its clinical validity. In our hospital, 110 patients with gynecologic cancer were registered. The prescribed dose was 50.4 Gy/28 fr. A DL-based three-dimensional dose prediction model was first trained by the dose distribution and structure data of whole pelvic VMAT (*n* = 100) created on the Monaco treatment planning system (TPS). The structure data of the test data (*n* = 10) were then input to RatoGuide, and RatoGuide predicted the dose distribution of the whole pelvic VMAT plan (PreDose). We established deliverable plans with Monaco and Eclipse TPS (DeliDose) based on PreDose and vendor-supplied optimization objectives. Medical physicists then manually developed plans (CliDose) for the test data. Finally, we evaluated and compared the dose distribution and dose constraints of PreDose, DeliDose, and CliDose. DeliDose, in both Eclipse and Monaco, was comparable to PreDose in most Dose constraints, planning target volume (PTV) coverage, and Dmax of the bladder, rectum, and bowel bag were better for DeliDose than for PreDose. Additionally, DeliDose demonstrated no significant difference from CliDose in most dose constraints. The blinded average scores of radiation oncologists for DeliDose and CliDose were 4.2 ± 0.4 and 4.3 ± 0.5, respectively, in Eclipse, and 4.0 ± 0.6 and 3.9 ± 0.5, respectively, in Monaco (5 is the max score and 3 is clinically acceptable). We indicated that RatoGuide can eliminate variations in plan quality between hospitals in whole pelvic VMAT irradiation and help develop VMAT plans in a short time.

**Supplementary Information:**

The online version contains supplementary material available at 10.1038/s41598-025-99717-y.

## Introduction

Intensity-modulated radiation therapy (IMRT) is a prevalently used radiotherapy technology in clinical situations. IMRT delivers doses to the target tumor tissue while avoiding various normal organs- organs at risk (OAR), and is used in a variety of treatment areas, including the brain^1^, head and neck^2^, esophageal^3^, prostate^4^, and gynecologic cancers^5^. IMRT technology has been more effective than three dimensional conformal radiation therapy(3DCRT) in terms of target coverage, dose uniformity, and toxicity reduction to normal organs^6^. The IMRT planning optimization method utilizes inverse planning, suggesting that after the physicist manually sets up the target and OAR dose parameters, the treatment planning system (TPS) independently calculates data such as the irradiation dose-monitor units (MUs) and irradiation field shapes for each of the various linear accelerator gantry angles. Although it may seem like an easy process, the quality of IMRT plans exhibits difficulty in ensuring uniformity, with time-consuming and costly planning. Dose parameter determination for targets and OARs depends on the user’s skill, so if the dose parameters are set unreasonably, thereby eventually creating a poor dose distribution. Therefore, the quality of the plans varies depending on the planners’ skills^7^. Additionally, skilled planners create a plan in a short period of time while less skilled planners take longer to identify the best dose parameters in terms of optimization time. Several researchers have developed systems for dose distribution prediction using deep learning (DL) to solve these problems^8^. Song et al.^9^ and Kajikawa et al.^10^ realized the research on rectal cancer and prostate cancer, respectively, through DL models, with dose distributions predicted from structure data and CT images. Xia et al.^11^ and Zhang et al.^12^ realized the automatic generation of deliverable plans for rectal cancer and prostate cancer, respectively, without human intervention. Early studies have focused on virtual dose predictions; however, recent efforts have advanced to the automated development of deliverable treatment plans with minimal human input.

Radiotherapy plays an important role in gynecologic cancer treatment, and effective survival rates have been achieved^13^. IMRT has emerged as an advanced technique, offering improved dosimetric properties and better clinical outcomes than conventional methods (3DCRT)^14–16^, has been widely used clinically this years.

Gronberg et al. reported DL-based dose distribution prediction of gynecologic tumors^17^. They predicted radiation oncologists’ acceptable dose distributions using a 3D dose prediction DL model. However, they only predicted dose distributions but did not establish deliverable plans. To the best of our knowledge, no study has reported DL-based volumetric arc radiation therapy (VMAT) dose distribution prediction to create deliverable plans for patients with gynecologic cancer.

Recently, a prototype artificial intelligence (AI)-based automated planning support system (RatoGuide [formerly known as AIVOT], AiRato Inc.) has been developed. In this system, the DL model predicts the VMAT dose distribution by inputting the structure datas into the DL model. The vendor provided objective functions to reproduce the AI dose distribution on the TPS. This enables us to create deliverable plans based on the dose distribution predicted by the DL model. This RatoGuide has already been reported to establish a good delivery plan in VMAT plans for patients with prostate cancer^18^. In this study, we used this RatoGuide to create VMAT deliverable plans with two TPSs (Eclipse [Varian Inc.], Monaco [Elekta Inc.]) of patients with gynecologic disorders with whole pelvic irradiation and clarify its dose accuracy.

The two objectives of the study were:

1. To generate a whole pelvic deliverable VMAT plan for patients with gynecologic disorders using a DL-based dose prediction system (RatoGuide), and to evaluate its clinical validity.
2. To verify the validity of the predicted results across different TPS. RatoGuide uses the Monaco dose distribution as the training data for the DL model; thus, the predicted dose distribution assumes creation with Monaco. We aimed to determine the possibility of Monaco to predict dose distribution on a different TPS, Eclipse.

## Materials and methods

### Patient data information

The training dataset on RatoGuide registered 100 patients with gynecological diseases who were treated with whole pelvic irradiation with VMAT at our hospital from 2018 to 2022. This study prepared a separate test dataset of ten patients with gynecological diseases treated with whole pelvic VMAT at our hospital in 2023.

The irradiation range included not only the pelvic region but also the para-aortic lymph nodes due to para-aortic lymph node metastasis. Of the 100 cases in the training set, 18 were irradiated up to the para-aortic lymph nodes, and of the 10 cases in the test set, 2 were irradiated up to the para-aortic lymph nodes.

All patients were scanned by planning computed tomography (CT) on a SOMATOM Definition AS+ (Siemens. Munich, Germany). The tube voltage was 120 kV, slice thickness was 2 mm, and pixel size was 1.2695 mm^2^. 

All experiments were performed in accordance with relevant institutional and national guidelines and regulations. In addition, the research design, data collection and management protocols, and scientific rationale of this study were approved by the Ethics Committee of Tohoku University Hospital. Given the retrospective nature of this study and the fact that no samples were obtained from human bodies, the requirement for informed consent was waived by the Ethics Committee of Tohoku University Hospital.

### Contouring

Radiation oncologists delineated tumor targets and OARs in all of the training and test datasets. The radiation oncologists contoured the primary tumor, the surrounding uterus and tissue, which was defined the clinical target volume (CTV) primary, in the target. The lymph node metastasis was delineated and a 5-mm margin was added to patients with lymph node metastasis to create a CTV nodal. CTV sub-volume was delineated as a precautionary zone. CTV primary was expected to significantly move depending on the degree of urine retention and bladder contraction; thus, a margin of 5–15 mm was added in six directions for planning target volume (PTV) primary. A PTV margin of 5–7 mm was added to the CTV sub-volume as a PTV sub-volume. Finally, PTV primary, nodal, and sub-volume were combined to create the target PTV.

The rectum, bladder, bowel bag, pelvic bone, and femoral joint were delineated for OAR. Additionally, the structure of the overlap between PTV and bowel bag was established and designated as OL_PTV_Bowel in this study.

Supplementary Table 1 presents the contouring data included for the training and test patients. Both the training and test datasets included various patients, ranging from those with a PTV confined to the pelvis to those with a more extensive PTV that extends into the upper abdomen.

### AI prediction and creation of deliverable dose distribution

#### Overall workflow for the creation of deliverable dose distribution

**Fig. 1:**
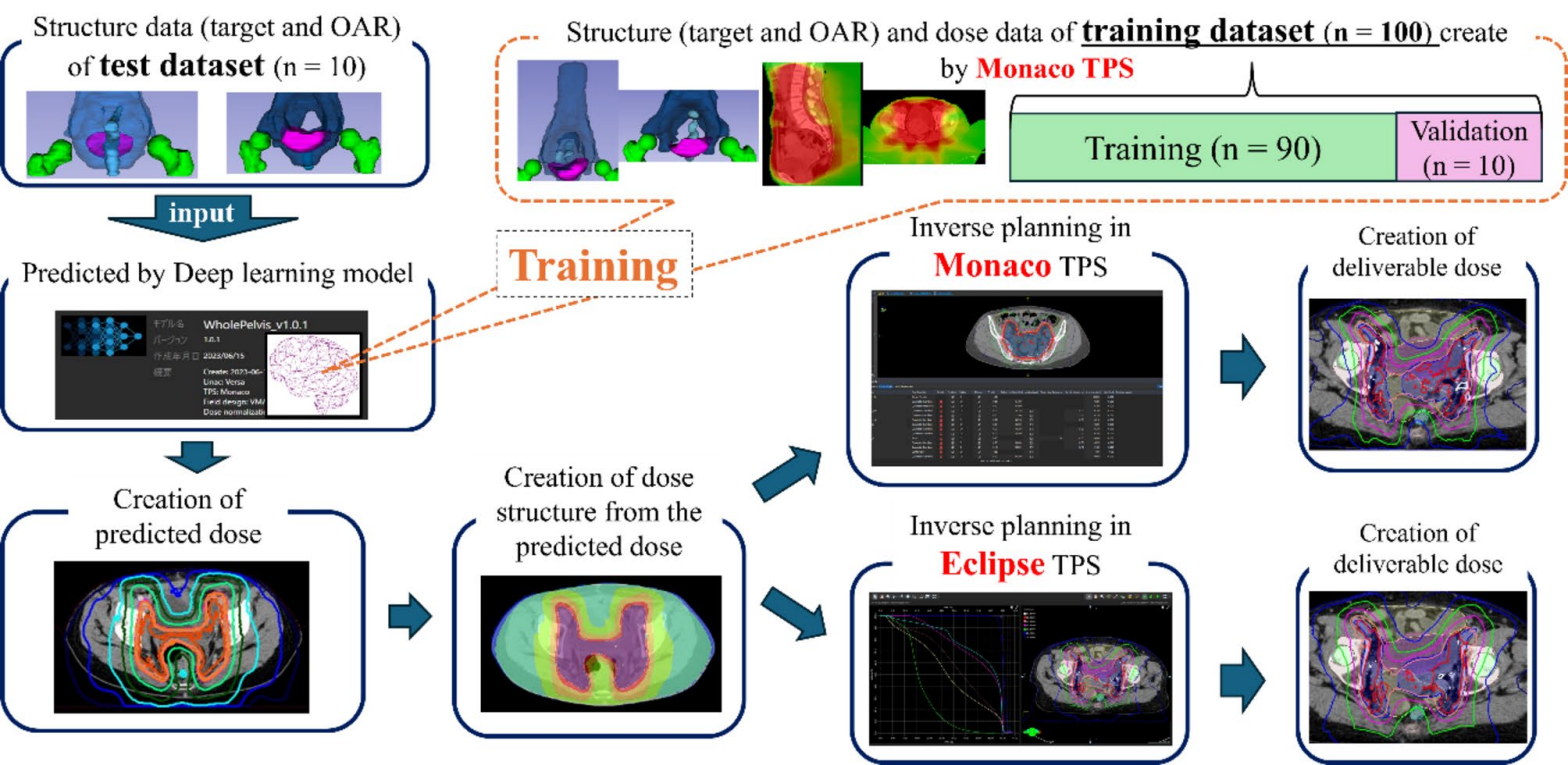
Study workflow. First, we created dose distributions for whole pelvic irradiations of volumetric arc radiation therapy (VMAT) for 100 patients in Monaco as a training dataset for RatoGuide. Second, we trained a deep-learning model of RatoGuide that would predict the VMAT dose distribution from the structure data. Third, we input the structure data of the 10 test dataset patients into this trained model, and the RatoGuide predicted the dose distribution. The predicted dose distributions were structured (dose structure) and imported into the treatment planning systems (Eclipse and Monaco). Inverse planning was performed on the treatment planning systems based on the dose structure to create the final deliverable dose distribution.

Figure 1shows the workflow diagram of this study. A previous paper reported by our research group described the detailed AI-based prediction and automated planning workflow of RatoGuide^18^. Training data set, which includes DICOM CT images, structure set, and dose distribution has been recreated for whole pelvic irradiations with VMAT of 100 patients in clinical protocol on Monaco. These data were input to RatoGuide for training the DL model of RatoGuide, which can predict the VMAT dose distribution from the structure data. The structure data of the 10 independent test dataset patients were then input to this trained model, and the RatoGuide predicted the dose distribution, named PreDose. The predicted dose distributions were structured (dose structure) and then imported into the TPS (Eclipse and Monaco). Inverse planning was performed on the TPSs based on the dose structure to create the final deliverable plan, the dose distribution of which was referred as DeliDose. The following sections describe this workflow in more detail.

#### Creation of training data and DL-based model training of RatoGuide

Structure data and dose distributions of VMAT are required for the DL-based model training in RatoGuide. To prepare the training dataset, we recreated whole pelvic VMAT plans for 100 patients under a same clinical protocol. The TPS was Monaco, and the prescribed dose of 50.4 Gy in 28 fractions was applied to the 50% volume of PTV, normalizing at D50. The dose constraints are based on the Japan Clinical Oncology Group (JCOG) 1402 (https://jrct.niph.go.jp/). Supplementary Table 2 presents the dose constraints of JCOG1402. The 3D dose distribution prediction model of RatoGuide for whole pelvic irradiation was dense dilated (DD) U-Net. The learning rate was 0.0001, the bench size was 4, and the voxel size was 3 × 3 × 3. The structure of the model was based on the method of Gronberg et al^19^. Further details of the inference, postprocessing, and modeling methods are kept confidential by the vendor which are not accessible. The input structures were PTV, bladder, bowel bag, rectum, femoral joint, pelvic bone, and body. Ninety and 10 patients were used for training and validation, respectively, and the losses of validation patients were monitored to ensure that they did not increase. Finally, a model was completed by training the DD U-Net on the dose and structure data. The completed model will predict the dose distribution for whole pelvic VMAT plans by inputting the structures (target and OAR). Furthermore, the predicted dose distribution will be output according to the set prescription dose (i.e., D50 PTV).

#### PreDose creation and structuring with RatoGuide

We input the structure data of 10 test data patients into the trained DD U-Net in RatoGuide to predict the VMAT dose distribution, which is defined as the PreDose in this study. To produce a clinical deliverable plan, optimization at TPS is required so that it can actually deliver the dose distribution. Therefore, the dose structures were developed in RatoGuide under the PreDose, which involved not only the isodose structure of the dose distribution in increments of 5–20% but also the overlapping region between the isodose dose structure and OARs to further decrease the OAR doses in the subsequent inverse planning. Supplementary Table 3 shows all dose structures. All dose structures could be created instantly using the templates in RatoGuide, Supplementary Fig. 1 presents the detailed view and list of those templates. Supplementary Fig. 2 presents the workflow of creating the dose structure. All dose structures were used for inverse planning of the TPS afterward.

#### Deliverable plan creation in Eclipse and Monaco

In this section, we discuss the process of turning a PreDose into an actual deliverable plan in TPS. We imported dose structures of the test dataset into TPS Eclipse and Monaco. The treating machines used were Versa model from Elekta and TrueBeam model from Varian because both of them have a radiation field size of 40 × 40 cm, which is adequate for patients undergoing whole pelvic radiation therapy. In Eclipse, the beam setting was two arcs, the calculation algorithm was AcurosXB (Version:16.1), the energy was 10 MV X-rays, and the collimator angle was 10° for the first arc and 350° for the second arc. Eclipse does not optimize well when the X-jaw is wide. Therefore, we manually closed the X-jaw of the first and second arcs slightly. The normal tissue objective (NTO) tool was not used because we were focusing on the high-dose region in PTV rather than the middle- or low-dose region, which has a highest priority among all parameters when NTO is applied and would not produce the desired dose distribution. In Monaco, the beam setting was two arcs, the calculation algorithm was Monte Carlo, the energy was 10 MV X-rays, and the collimator angle was 5° for both the first and second arcs. Inverse planning was performed based on the optimization parameters provided by the vendor, AiRato company, which produced RatoGuide. Supplementary Table 4 and 5 present the vendor-provided optimization parameters inputting the constraining parameters to the dose structure of the AI prediction created in the previous section, the dose distribution was reproduced in TPS. The provided optimization parameters were created based on following concept:

First priority: The 95% dose covers as much of the PTV as possible.

Second priority: The dose constraints of JCOG1402 are met (must meet the tolerances).

Third priority: The dose distribution to the rectum, bladder, and bowel bag reproduces the AI prediction as much as possible.

Fourth priority: The medium and low-dose distributions to the body outside the PTV are as similar to the AI prediction as possible.

Fifth priority: The hotspot within the PTV should be as small as possible.

The optimization parameters were set to be versatile so that they could be applied to any patient. Optimization could be performed only once to create the final deliverable plan based on these optimization parameters. This study defined the dose distribution of the deliverable plan known as DeliDose.

### Clinical plan creation

To evaluate the clinical relevance of the DeliDose plan, a clinical plan was developed using the conventional method (manual plan creation) without any use of AI. The dose distribution of these plans were then compared with that of DeliDose in the test dataset. As the clinical used plans of patients in this study had followed our institution’s protocol, which differed from JCOG 1402 (this study protocol), the clinical plan were recreated by one medical physicist according to JCOG 1402 for this study. The clinical plan settings (number of Arcs, calculation algorithm, energy, and collimator angle) for Eclipse and Monaco were same as DeliDose plans. The optimization goal was to minimize the OAR dose while maintaining target coverage. The number of optimization was unlimited until the medical physicist determined that the OAR could not be further reduced while maintaining the target dose coverage. The dose distribution of this final clinical plan was defined as CliDose in this study.

### Evaluation method

We evaluated the clinical validity of three dose distributions generated in 10 test dataset patients: AI-predicted dose distribution (PreDose), AI-predicted dose distribution made deliverable dose (DeliDose), and dose distribution manually generated by a medical physicist (CliDose).

Firstly, dose volume histogram (DVH) parameters were compared and *t*-tests were performed for the three plans following the JCOG1402 dose constraints.

Secondly, dice coefficients were then used to evaluate the agreement in dose distribution (20% dose intervals) of PreDose vs. DeliDose and DeliDose vs. CliDose. Specifically, PreDose reproduction on the TPS was evaluated by assessing the degree of agreement between the PreDose and DeliDose dose distributions every 20%. Additionally, the DeliDose was compared with the CliDose by evaluating the degree of agreement between the DeliDose and CliDose.

Thirdly, the gamma passing rates (GPR) were then measured using a 3D diode array detector (ArcCheck, SunNuclear Melbourne, FL, USA) to evaluate the DeliDose as a clinically deliverable plan. The setting criterion was 3%/2 mm and the dose threshold was 10%, as the American Association of Physicists in Medicine Task Group (AAPM-TG) 218 report recommended^20^. Additionally, the GPR of the CliDose was measured for comparison.

Fourthly, we measured the modulation complexity score (MCS) to assess the use of unreasonable motion or extremely small irradiation fields in the VMAT plan’s multi-leaf collimator (MLC)^21,22^.

We calculated MCS using RatoGuide and calculated dice coefficients with Eclipse. The Wilcoxon rank sum test was used to test the significance of the dose volume histogram (DVH) parameters. Bonferroni-corrected *p*-values were used to assess significant differences in DVH parameters between PreDose and DeliDose, DeliDose and CliDose, and PreDose and CliDose. Statistical significance level of 0.05/15 ≈ 0.0033 was used. The number of DVH parameters of the dose constraint was set to 15. MATLAB (Math, Inc.) was used to analyze significance tests.

Finally, a senior radiation oncologist visually evaluated the dose distributions of all testset plans and scored them on a scale of 1–5 quality increments, with 5 being excellent, 4 being close to perfect with one or two improvements, 3 being clinically acceptable (which means the plan can irradiate to patients), 2 being not acceptable for irradiation to the patients, and 1 being poor. Supplementary Table 6 presents the details of the way to score in radiation oncologist evaluation.

## Results

### Comparison of dose constraints

Tables 1 and 2 summarize the DVH metrics for PreDose, DeliDose, and CliDose of the 10 test dataset patients.

**Table 1:** DVH metrics for DeliDose, CliDose, and PreDose in Monaco TPS.

Monaco DVH parameters							
Structure name	DVH metric	PreDose	DeliDose	CliDose	P-value (PreDose vs. DeliDose)	P-value (DeliDose vs. CliDose)	P-value (PreDose vs. CliDose)
		Mean ± SD	Mean ± SD	Mean ± SD			
Body	Dmax (%)	106.5 ± 0.1	109.7 ± 0.8	108.7 ± 0.5	**< 0.001**	0.014	**< 0.001**
PTV	D50% (%)	99.9 ± 0.0	100.0 ± 0.0	100.0 ± 0.2	**0.001**	0.582	**0.001**
	D98% (%)	93.2 ± 0.5	93.8 ± 0.4	93.9 ± 0.4	0.011	0.623	0.005
	D95% (%)	94.9 ± 0.4	95.5 ± 0.4	95.7 ± 0.4	0.008	0.256	**0.002**
	D2% (%)	103.2 ± 0.3	104.4 ± 0.5	103.9 ± 0.3	**< 0.001**	0.021	**0.001**
OL_PTV_Bowel	Dmax (%)	106.5 ± 0.1	104.4 ± 0.8	105.0 ± 0.9	**< 0.001**	0.198	**0.002**
Rectum	V40Gy (%)	58.7 ± 15.6	57.9 ± 15.5	59.1 ± 15.4	0.791	0.850	0.791
	V50Gy (%)	6.8 ± 5.1	10.0 ± 6.4	14.4 ± 7.3	0.212	0.307	0.019
	Dmax (%)	105.4 ± 0.7	103.7 ± 0.6	104.0 ± 0.6	**< 0.001**	0.427	**0.002**
Bladder	V45Gy (%)	46.9 ± 10.1	51.2 ± 11.2	46.3 ± 10.0	0.385	0.307	0.850
	Dmax (%)	106.3 ± 0.3	104.2 ± 1.0	104.8 ± 0.5	**< 0.001**	0.140	**< 0.001**
Bowel Bag	V40Gy (%)	23.1 ± 5.3	22.6 ± 5.4	23.9 ± 5.3	0.496	0.273	0.385
PelvicBones	V10Gy (%)	89.7 ± 2.3	88.1 ± 2.9	86.0 ± 2.1	0.273	0.064	0.006
	V40Gy (%)	27.6 ± 2.6	28.6 ± 2.9	28.8 ± 3.0	0.345	0.940	0.326
Femoral Joint	V30Gy (%)	3.5 ± 2.6	3.9 ± 2.9	5.9 ± 4.6	0.734	0.345	0.241

Dmax: maximal dose; D50%: dose administered to 50% of volume; D98%: dose administered to 98% of volume; D95%: dose administered to 95% of volume; D2%: dose administered to 2% of volume; PTV: planning target volume; OL_PTV_Bowel: common area of PTV and Bowel Bag.

**Table 2:** DVH metrics for DeliDose, CliDose, and PreDose in Eclipse TPS.

Eclipse DVH parameters							
Structure name	DVH metric	PreDose	DeliDose	CliDose	P-value (PreDose vs. DeliDose)	P-value (DeliDose vs. CliDose)	P-value (PreDose vs. CliDose)
		Mean ± SD	Mean ± SD	Mean ± SD			
Body	Dmax (%)	106.5 ± 0.1	108.9 ± 0.9	107.7 ± 0.4	**< 0.001**	0.011	**< 0.001**
PTV	D50% (%)	99.9 ± 0.0	100.0 ± 0.0	100.0 ± 0.0	**< 0.001**	1.000	**< 0.001**
	D98% (%)	93.2 ± 0.5	94.8 ± 0.4	95.1 ± 0.2	**< 0.001**	0.076	**< 0.001**
	D95% (%)	94.9 ± 0.4	96.4 ± 0.4	96.4 ± 0.2	**< 0.001**	0.496	**< 0.001**
	D2% (%)	103.2 ± 0.3	104.1 ± 0.2	103.6 ± 0.1	**< 0.001**	**0.001**	**0.001**
OL_PTV_Bowel	Dmax (%)	106.5 ± 0.1	104.8 ± 1.3	105.0 ± 1.3	0.026	0.734	0.075
Rectum	V40Gy (%)	58.7 ± 15.6	61.2 ± 15.6	65.1 ± 11.8	0.571	0.623	0.473
	V50Gy (%)	6.8 ± 5.1	7.4 ± 6.8	11.0 ± 7.7	0.910	0.212	0.212
	Dmax (%)	105.4 ± 0.7	102.4 ± 0.8	102.7 ± 0.8	**< 0.001**	0.473	**< 0.001**
Bladder	V45Gy (%)	46.9 ± 10.1	52.4 ± 11.2	51.1 ± 8.4	0.186	0.850	0.212
	Dmax (%)	106.3 ± 0.3	102.7 ± 1.1	103.0 ± 0.7	**< 0.001**	0.384	**< 0.001**
Bowel Bag	V40Gy (%)	23.1 ± 5.3	22.9 ± 5.5	23.5 ± 5.1	0.734	0.623	0.850
PelvicBones	V10Gy (%)	89.7 ± 2.3	88.1 ± 2.9	85.5 ± 1.8	0.345	0.031	**0.002**
	V40Gy (%)	27.6 ± 2.6	30.7 ± 3.1	26.8 ± 2.2	0.026	0.009	0.345
Femoral Joint	V30Gy (%)	3.5 ± 2.6	6.3 ± 4.3	2.0 ± 1.1	0.104	0.007	0.257

Dmax: maximal dose; D50%: dose administered to 50% of volume; D98%: dose administered to 98% of volume; D95%: dose administered to 95% of volume; D2%: dose administered to 2% of volume; PTV: planning target volume; OL_PTV_Bowel: common area of PTV and Bowel Bag.

### PreDose vs. DeliDose

DeliDose provided better dose coverage for both Monaco and Eclipse than PreDose for target coverage, such as PTV D95% (*p* = 0.08 for Monaco, *p* < 0.001 for Eclipse) and D98% (*p* = 0.011 for Monaco, *p* < 0.001 for Eclipse), indicating a typical 95% and 98% volume of the target irradiated dose. DeliDose achieved the same dose reduction as PreDose for both Eclipse and Monaco for OARs. Additionally, it reduced the OAR doses more than PreDose for the maximum radiation dose (Dmax) of the rectum and bladder. However, some DVH metrics (volume irradiated with 45 Gy dose: V45 Gy for the bladder and volume irradiated with 50 Gy dose: V50 Gy for the rectum) of DeliDose resulted in higher doses than PreDose.

### DeliDose vs. CliDose

Most DVH metrics shown the same level between DeliDose and CliDose in Monaco. Most DVH parameters had no significant difference between DeliDose and CliDose in Eclipse; however, the PTV D2% was significantly lower with CliDose than it with DeliDose in Eclipse (*p* = 0.001).

However, the dose reduction was greater with DeliDose than with CliDose in terms of V40 Gy (DeliDose: 61.2%, CliDose: 65.1% for Eclipse) and V50 Gy (DeliDose: 7.4%, CliDose: 11.0%) for the rectum, although the difference did not reach statistical significance.

**Fig. 2:**
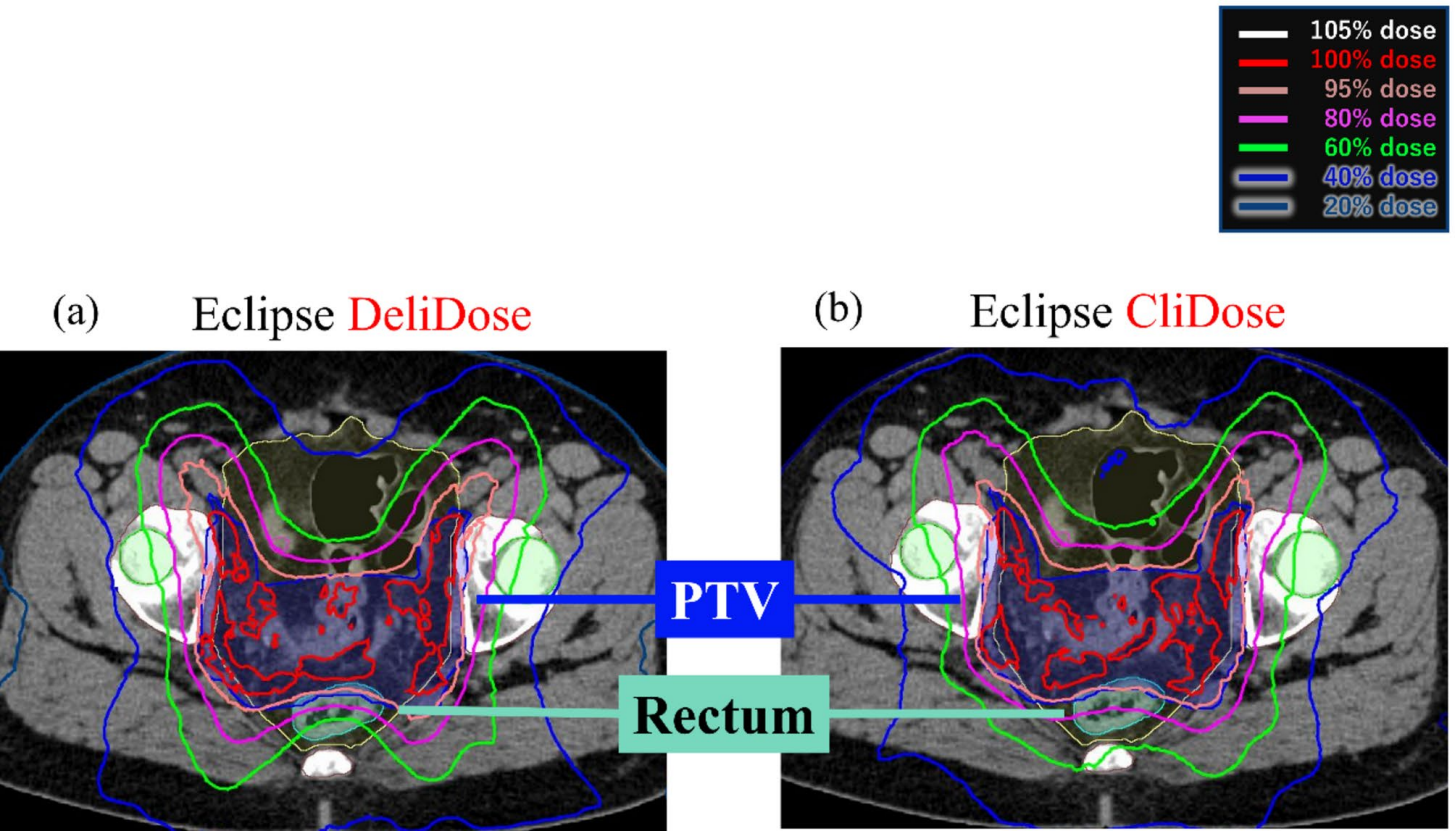
Comparison of DeliDose and CliDose in Eclipse.

Figure 2 shows one typical example where the rectal dose was lower with the DeliDose than with the CliDose.

Figure 2a shows that the DeliDose consistently lowered the high to medium rectal dose region, whereas Fig. 2b presents that CliDose was unable to reduce the high to medium dose region in the rectum.

### Comparison of DVH curves

**Fig. 3:**
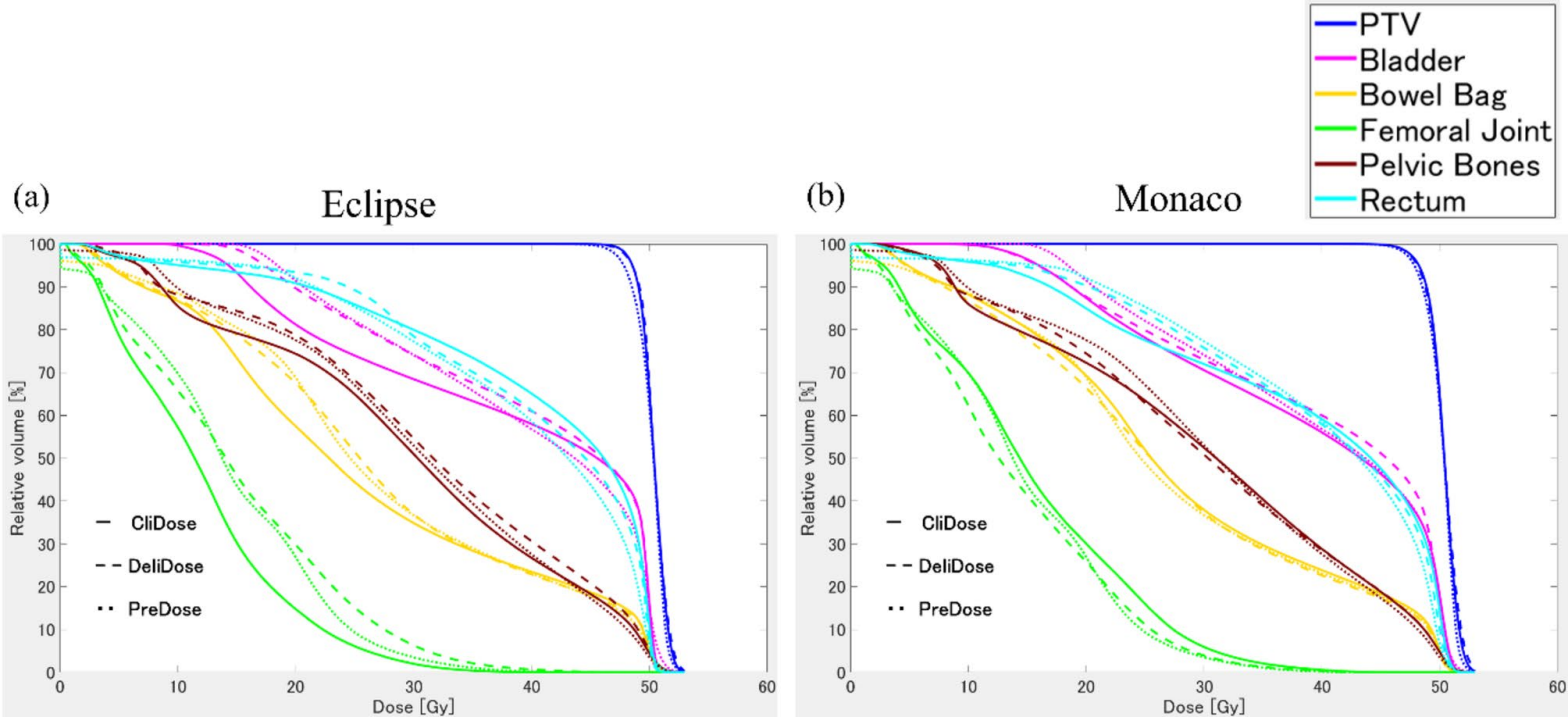
Dose volume histogram (DVH) curves of organ at risk (OAR) and planning target volume (PTV) for PreDose, DeliDose, and CliDose of Eclipse (a) and Monaco (b).

Figure 3 shows the OAR and PTV DVH curves for PreDose, CliDose, and DeliDose of Eclipse and Monaco.

The OAR and target DVH of the DeliDose and PreDose were in good agreement in Eclipse. The CliDose was lower than the DeliDose and PreDose at the low OAR doses. However, the CliDose dose was greater than that of the DeliDose only at the high doses of the rectum.

**Fig. 4:**
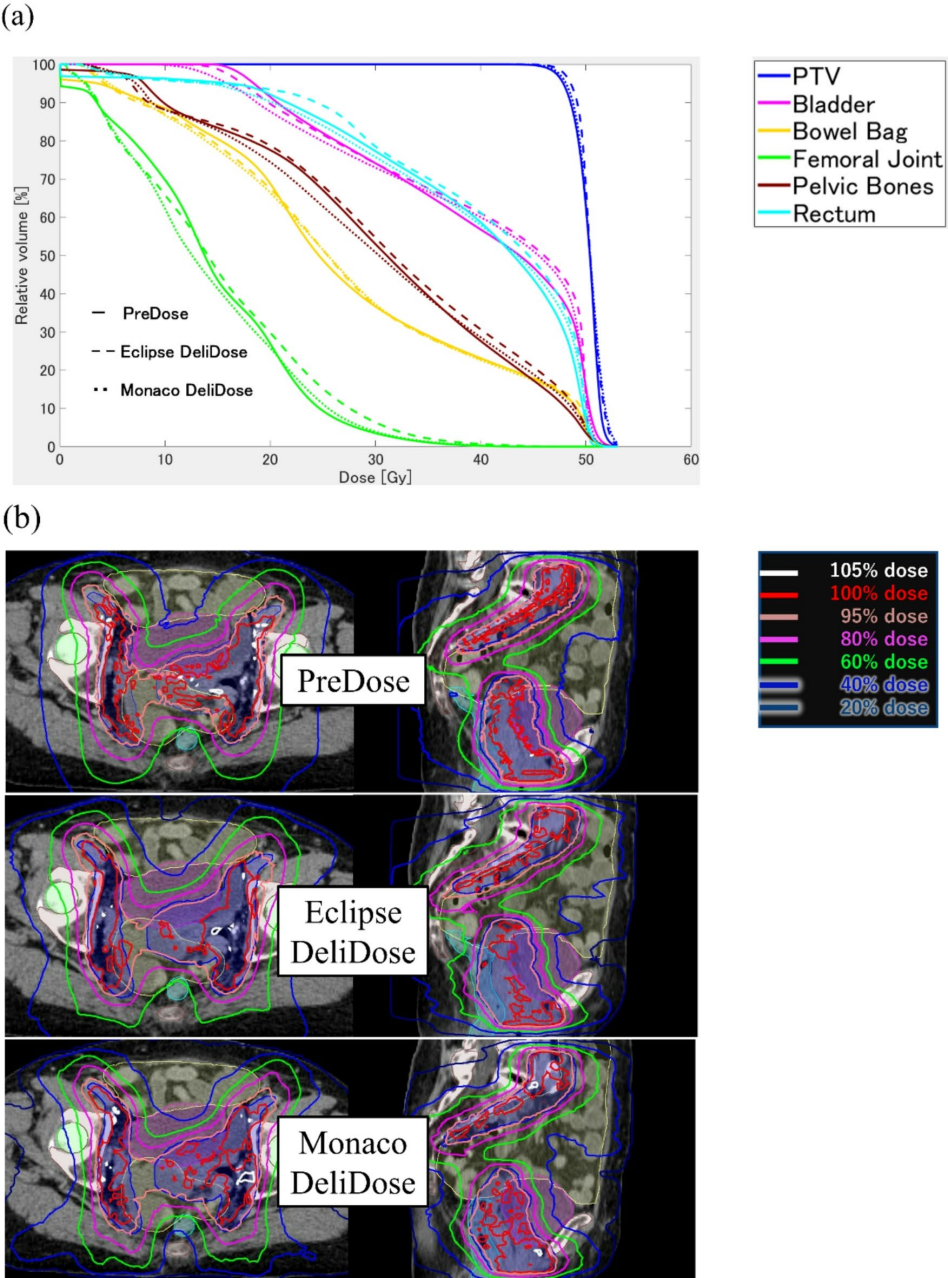
DVH curves for PreDose and DeliDose in Eclipse and Monaco for 10 patients (a) and dose distributions for PreDose and DeliDose of Eclipse and Monaco in one typical patient (b).

PreDose, CliDose, and DeliDose all demonstrated good agreement between OAR and target DVH in Monaco. Additionally, Fig. 4 focuses on the difference between DeliDose and PreDose in Eclipse and Monaco. Figure 4a illustrates that both Eclipse and Monaco have almost the same DVH as PreDose, indicating that both TPSs can adequately reproduce the AI dose distribution (PreDose). Furthermore, the dose distributions to the bladder, rectum, and pelvic bone were well reproduced by both Eclipse and Monaco in the PreDose on the dose distribution (Fig. 4b). One difference was that the low and medium doses to the body were distributed smoothly in PreDose, whereas Eclipse and Monaco demonstrated a jagged and spiky dose distribution.

### Evaluation of dose distribution agreement

Supplementary Table 7 shows the PreDose vs. DeliDose and CliDose vs. DeliDose dice coefficients for Monaco and Eclipse per 20% dose structure.

The mean dice coefficients of 0–20%, 20–40%, 40–60%, 60–80%, 80–100%, and 100–120% for PreDose and DeliDose in Monaco were 0.97 ± 0.01, 0.78 ± 0.03, 0.74 ± 0.02, 0.79 ± 0.01, 0.81 ± 0.02, and 0.71 ± 0.03, respectively. They were all > 0.7. The mean dice coefficients of the same dose structure for CliDose and DeliDose were 0.97 ± 0.01, 0.72 ± 0.03, 0.68 ± 0.02, 0.70 ± 0.02, 0.79 ± 0.02, and 0.71 ± 0.03, respectively.

Furthermore, the mean dice coefficients of 0–20%, 20–40%, 40–60%, 60–80%, 80–100%, and 100–120% dose structure for PreDose and DeliDose in Eclipse were 0.98 ± 0.01, 0.82 ± 0.02, 0.78 ± 0.01, 0.78 ± 0.02, 0.78 ± 0.03, and 0.67 ± 0.03, respectively. The dice coefficient was > 0.8 in the averaged overall 20% interval dose structures. The dice coefficients of the same dose structure for CliDose and DeliDose were 0.97 ± 0.01, 0.75 ± 0.05, 0.69 ± 0.07, 0.69 ± 0.06, 0.79 ± 0.03, and 0.75 ± 0.07, respectively.

**Fig. 5:**
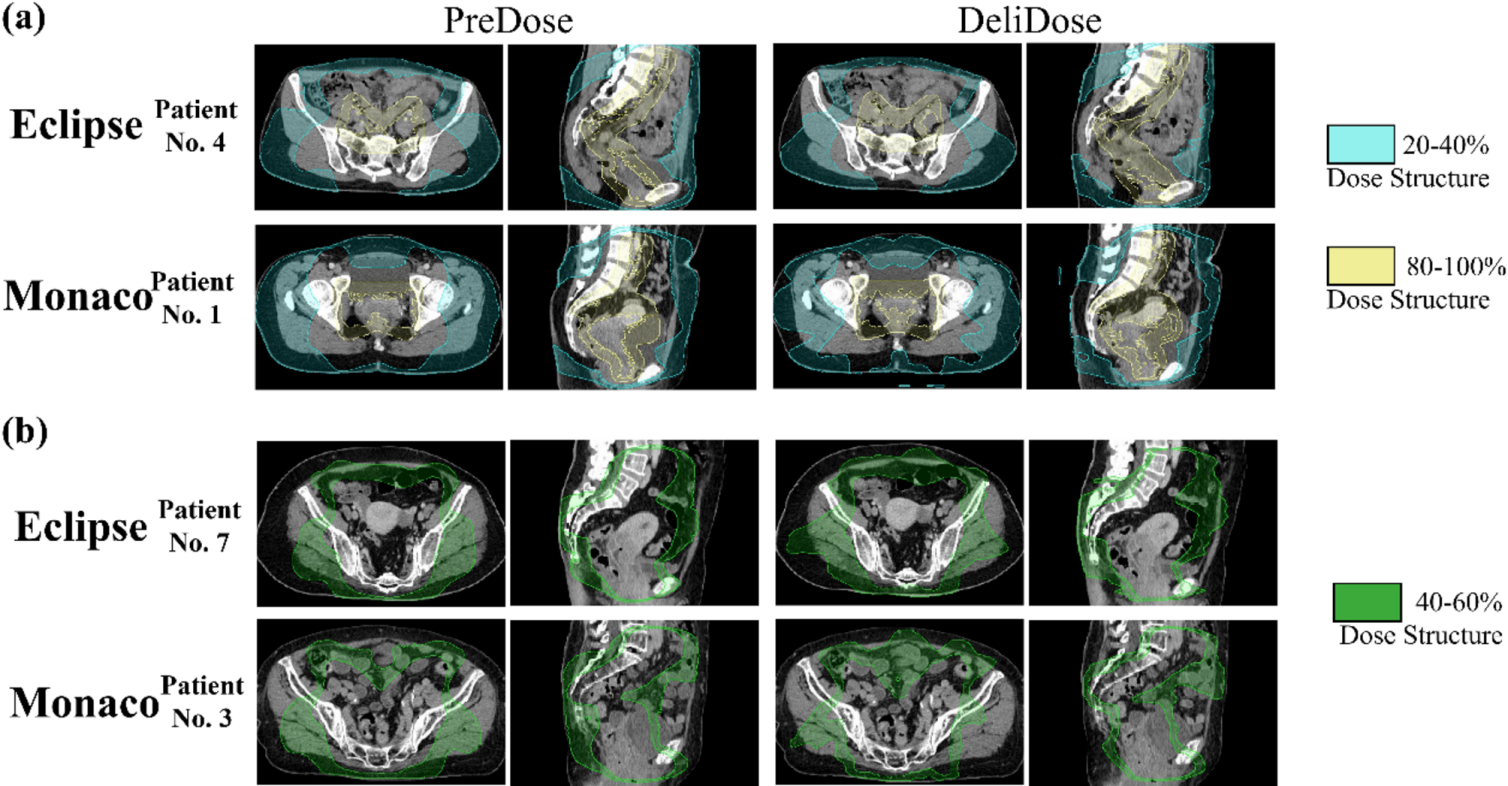
Dose structure of PreDose and DeliDose in patients where the dice coefficients of both were well (a) and poor (b) matched.

Figure 5 shows patients with good (Fig. 5a) and poor (Fig. 5b) agreement between the PreDose and DeliDose dose structure dice coefficients.

Both Eclipse and Monaco exhibited high agreement between PreDose and DeliDose for the 20–40% and 80–100% dose structures, with the DeliDose reproducing the PreDose dose (Fig. 5a). Conversely, the 40–60% dose structure demonstrated worse agreement between PreDose and DeliDose than the other dose structures (Fig. 5b). PreDose was more concentrated at the 40% dose, whereas DeliDose was less concentrated at the 40% dose, causing slightly worse values for the dice coefficient for the 40–60% dose structure.

### Radiation oncologist’s scores

The radiation oncologists’ average scores for DeliDose and CliDose were 4.2 ± 0.4 and 4.3 ± 0.5 in Eclipse and 4.0 ± 0.6 and 3.9 ± 0.5 in Monaco, respectively. All DeliDose scores in both Monaco and Eclipse were ≥ 3, indicating their clinical utility.

**Fig. 6:**
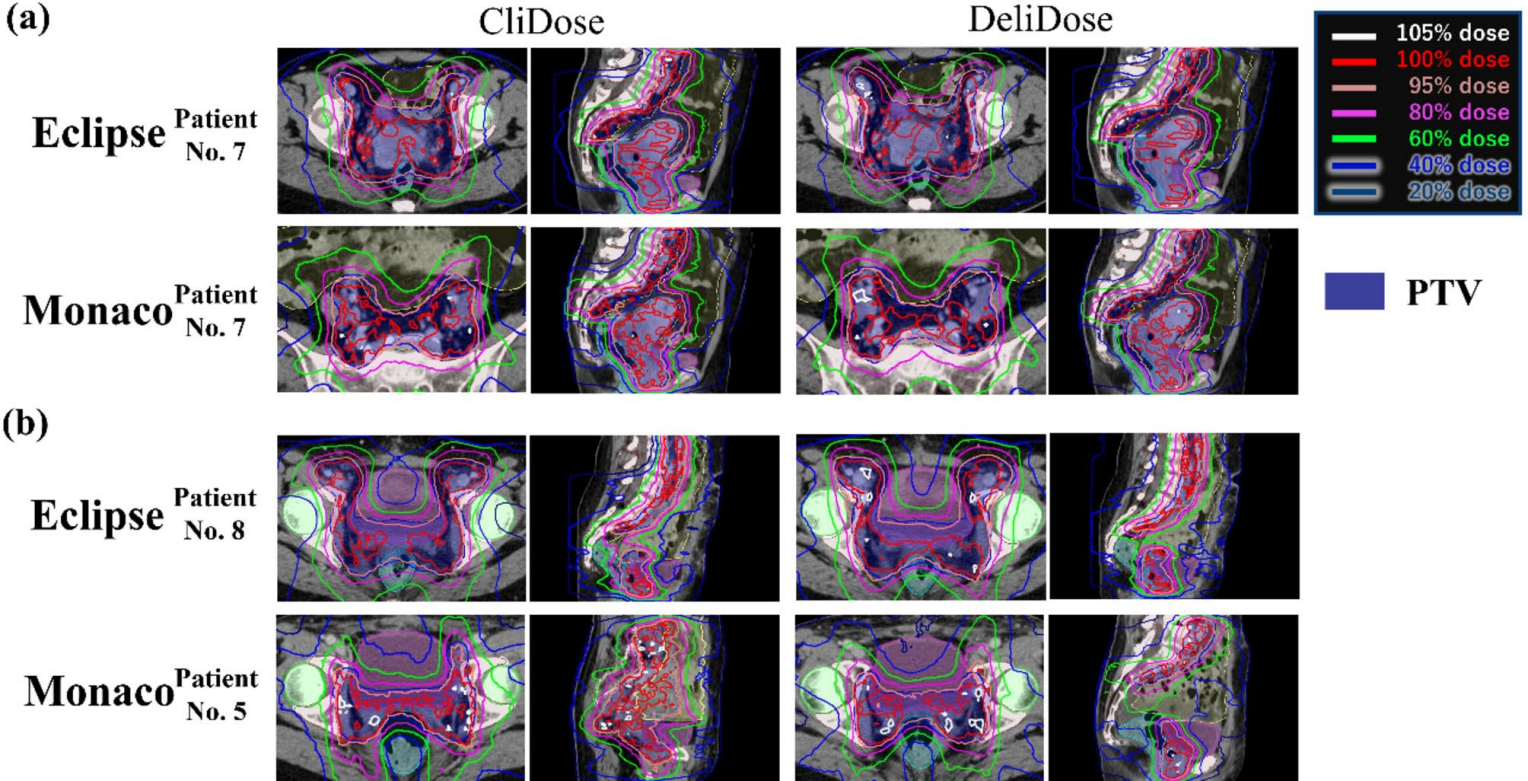
Dose distributions for typical cases with good (a) and poor (b) DeliDose radiation oncologist scores in Eclipse and Monaco.

Figure 6a shows dose distribution for typical cases with good DeliDose radiation oncologist scores in Eclipse (score: DeliDose 5.0 vs. CliDose: 5.0) and Monaco (score: DeliDose: 5.0 vs. CliDose: 4.0). Figure 6a shows that DeliDose and CliDose in Eclipse demonstrated high scores because of good PTV coverage and low OAR dose. Figure 6a illustrates high scores for DeliDose in Monaco because the medium dose to the body was not extended. However, CliDose exhibited a lower score than DeliDose because of the slightly extended medium dose to the body outside the PTV.

Figure 6b presents dose distribution for typical cases with worse DeliDose radiation oncologist scores in Eclipse (score: DeliDose 4.0 vs. CliDose: 5.0) and Monaco (score: DeliDose: 3.0 vs. CliDose: 3.0). Figure 6b shows lower score in DeliDose than in CliDose of Eclipse because bladder and pelvic bone doses were not reduced. Figure 6b indicates lower scores for both DeliDose and CliDose in Monaco because of hotspots in the bowel and the medium dose that extended to the body outside the PTV. Radiation oncologist highlighted the bladder dose and bowel bag hotspot as concern area for DeliDose. We investigated the relationship between the overlap volume of patients with higher bladder dose and bowel bag hotspots for additional analysis. Supplementary Fig. 3 presents the correlation between the volume of the overlap between the PTV and OAR and the Dmax of the OAR of DeliDose. Particularly in Eclipse, the larger the volume of overlap between the PTV and bowel bag, the higher the Dmax of the bowel bag in DeliDose (*r* = 0.73). The same trend was observed for the bladder (*r* = 0.68).

### Investigating the versatility of DeliDose

To evaluate the versatility of the RatoGuide AI model and DeliDose plan, we created DeliDose plans for three additional patients of whose planning target was particularly longer and difficult,  including additionally the inguinal lymph node region. Patients with such targets were not included in the training data for the AI model. Supplementary Fig. 4 presents the results of the three patients; all of the plans met the dose constraints of JCOG1402. The average scores of the radiation oncologist for Monaco and Eclipse were 3.3 and 3.7, respectively; the scores for the three patients were ≥ 3 (clinically acceptable plan).

### GPR and MCS

The mean 3%/2 mm GPR for DeliDose and CliDose were 97.8%±1.5% and 97.4%±1.4% (Eclipse) and 95.1%±1.8% and 95.3%±1.4% (Monaco), and their mean MCS were 0.25±0.02 and 0.21±0.02 (Eclipse) and 0.13±0.01 and 0.12±0.01 (Monaco), respectively.

## Discussion

We created a DL-based deliverable VMAT whole pelvic treatment plan for patients with gynecologic cancer using RatoGuide and evaluated its clinical validity. While several DVH metrics of DeliDose were inferior to the clinical plan (CliDose) (Tables 1 and 2), DeliDose was comparable to CliDose without significant differences for most DVH metrics (Tables 1 and 2). Additionally, we used Monaco-generated VMAT dose distributions as training data and reproduced the predicted dose distributions well even with a different TPS, Eclipse (Supplementary Table 7: the mean dice coefficients on both TPS of which was 0.80: PreDose vs. DeliDose). We especifically revealed the possibility of creating deliverable dose distributions on the TPS that differ from the training dataset. RatoGuide can significantly improve the efficiency of planning in clinical practice for whole pelvic VMAT of gynecologic cancers.

### PreDose vs. DeliDose

DVH curves of OAR and PTV for PreDose and DeliDose were in good agreement for both Eclipse and Monaco (Fig. 4). These results indicate the possibility of creating deliverable dose distributions at multiple TPSs by determining optimization parameters for each TPS from an AI model trained on dose distributions created at one TPS.

Kadoya et al. used RatoGuide to predict and establish deliverable VMAT plans for patients with prostate cancer^18^. They created deliverable plans for comparatively small-volume targets, the prostate, with RatoGuide predicting the dose distribution and reproducing it well in the Eclipse. We also well reproduced the dose distribution predicted by RatoGuide and created deliverable plans even for the large-volume target of whole pelvic irradiation. We indicated that RatoGuide possible create deliverable plans nearly automatically for targets with various volume sizes.

DeliDose in Monaco improved the D98% and D95% of PTV and the Dmax of the bladder and rectum compared to PreDose (Table 1). Similar improvements were observed in Eclipse (Table 2). This indicates that the TPS optimization parameters may improve some DVH metrics in areas where further improvements can be made to RatoGuide’s predictions. This indicates that you can create deliverable plans that suit your hospital if the AI dose distribution predicted by RatoGuide does not match the dose constraint policy of your institution by creating optimization parameters tailored to the dose constraints of your center.

Both Monaco and Eclipse showed that DeliDose significantly lowered the Dmax of the rectum and bladder compared with PreDose (*p* < 0.001 [Tables 1 and 2]). This is of great clinical significant because the radiation oncologist pay great attention to a low Dmax for OAR when scoring. In contrast, the maximum dose of Body and PTV was significantly smaller for PreDose than for DeliDose (*p* < 0.001 [Tables 1 and 2]). However, the maximum dose of DeliDose was located in the PTV, which is not related to the OAR. The mean values of Body Dmax in DeliDose were 109.7% ± 0.83% and 108.9% ± 0.93% for Monaco and Eclipse, respectively,which were not considered clinically problematic. The radiation oncologist also did not point out the maximum dose of Body and PTV, of these DVH parameters we think would not affect the scoring.

PreDose and DeliDose demonstrated relatively good agreement for every 20% dose structure (Supplementary Table 7). However, the dice coefficient for the 40–60% dose structure was lower than for the other dose structures, and medium doses, such as the 40% dose, were more predominant for DeliDose than for PreDose. PreDose predicts smooth and highly concentrated dose distribution for all patients, but DeliDose has difficulty in creating highly concentrated dose distribution for patients with large PTV volume, resulting in a 40% dose spread in some patients for DeliDose compared to PreDose. Gronberg et al. reported that AI predicts a smooth dose distribution for the medium and low dose range^17^, which is different from the actual deliverable dose distribution.

### DeliDose vs. CliDose

DeliDose and CliDose had no significant difference for most DVH parameters (Tables 1 and 2). These results indicate that in terms of OAR dose and target parameters, DeliDose achieved the almost same dose reduction and target coverage with a single optimization as CliDose, which the medical physicist had to redo several times. Medical physicist had to manually achieve optimal OAR dose reduction and target coverage because the creation of the CliDose started with no goal. Furthermore, the endpoint of the optimization process is completely determined by the medical physicist, making it difficult to make reliable quantitative decisions. Conversely, the AI provides the goal, the prediction dose distribution, of optimization with DeliDose; thus, we could have created a dose distribution that is comparable to that of CliDose, even with only one optimization.

However, CliDose was significantly better than DeliDose in terms of PTV D2% for Eclipse (*p* = 0.001, Table 2). This was the only DVH parameter that significantly differed between DeliDose and CliDose. Medical physicists redid the optimization several times to decrease the hotspots in CliDose, resulting in CliDose having fewer hotspots than DeliDose within the PTV. However, the average PTV D2% of DeliDose was 104.1% ± 0.2%, which is considered clinically acceptable. Conversely, the high and medium doses were lower with the DeliDose than with the CliDose in the rectum (Tables 1 and 2). The medical physicist who created the CliDose stated the difficulty of determining how much to reduce the rectal medium and high doses because PTV coverage would decrease if the rectal medium and high doses were reduced too much. Conversely, no variation was observed because DeliDose reduced the medium and high rectal doses without decreasing the PTV coverage consistently in all patients. Additionally, DeliDose may be a marker for clinically experienced planners as to how much to reduce the OAR dose and may indicate a role for quality assurance in the VMAT plan. Moreover, DeliDose produced a certain level of plans for all patients with gynecologic diseases, which means it is expected to equalize the plan quality.

### Radiation oncologist’s score

The radiation oncologist’s blinded scores were higher for CliDose in Eclipse, with an average score of 4.2 for DeliDose and 4.3 for CliDose.

DeliDose in Eclipse demonstrated lower scores than CliDose because of insufficient dose reductions in the bladder and the pelvic bone, as well as hot spots in the bowel bag. Supplementary Fig. 5 has shown the dose distribution for a typical case in Eclipse. DeliDose had more hotspots of 105% dose than CliDose.

Supplementary Fig. 3 suggests that this bowel bag hotspot occurs with a larger overlap volume between the PTV and bowel bag (correlation coefficient: *r* = 0.73). A few hotspots > 105% were observed in the PreDose. However, hotspots did occur in the process of reproducing the PreDose in Eclipse. The greater the overlap volume between the PTV and OAR, the more difficult it is to reproduce deliverable plan in TPS (i.e., the more difficult it is to create a DeliDose), and the greater the discrepancy between PreDose and DeliDose.

The average score for DeliDose in Monaco was 4.0, whereas the average score for CliDose was 3.9. DeliDose achieved a tolerance level of all dose constraints in both Eclipse and Monaco, and all patients had a score of ≥ 3, indicating that the DeliDose plans were clinically acceptable.

The overall score of Monaco was lower than that of Eclipse, the most predominant reason for which was the extension of the medium dose to the body outside the PTV. This may be because of the difference of TPS. AbuEmira et al. found that the Gradient Index is significantly larger for Monaco than for Eclipse^23^, which is consistent with our results. Eclipse’s VMAT plan has complex movement of the left and right MLCs, whereas Monaco’s VMAT plan has the left and right MLCs moving together at each angle of increment. This movement of Monaco’s MLC creates variations in the areas in which the dose continues to be irradiated and areas are not irradiated as much. Therefore, we considered it would be difficult to create a highly concentrated plan in Monaco. We considered that it is necessary to reduce the incremental angle to prevent this situation. According to Kang et al., the smaller the increment, the smaller the Gradient Index^24^. However, plan complexity may increase because of the increase in MUs, which may result in failed GPR measurements and extend treatment time.

### Investigating the versatility of DeliDose

To evaluate the versatility of the AI model and DeliDose, we created DeliDose in three additional patients with targets including the inguinal lymph node region (Supplementary Fig. 4). All plan of three patients met the dose constraints, and the scores of the radiation oncologist were ≥ 3, indicating that these DeliDose plans were clinically acceptable. The RatoGuide AI model was trained on patients with various size of targets, ranging from normal whole pelvis region to those with long cephalad directions to para-aortic lymph node (Supplementary Table 1). Therefore, the AI model would have been able to cope with the unknown data to some extent for similar treatment region. Furthermore, even if the AI model predicts a dose distribution that is difficult to reproduce, DeliDose can adjust it using the optimization parameters. We believe that we have developed a highly versatile DeliDose plan that can be applied to any patient by optimizing the PTV with higher priority than the OAR and PreDose structures (Supplementary Tables 4 and 5).

However, further optimized DeliDose plans can be developed by creating optimized multiple optimization parameters and multiple AI models for patient-specific OARs and PTVs. Therefore, we believe that creating even better AI-deliverable plans will be possible by evaluating the prediction error of the AI model and using different AI models or optimization parameters for patients with large prediction errors.

### GPR and MCS

The mean 3%/2 mm GPR value for DeliDose and CliDose were 97.8% and 97.4% in Eclipse and 95.1% and 95.3% in Monaco, respectively. DeliDose and CliDose demonstrated no marked difference in GPR value, indicating that the DeliDose plan is completely deliverable. The mean MCS value for DeliDose and CliDose were 0.25 and 0.21 in Eclipse and 0.13 and 0.12 in Monaco, respectively. Both CliDose and DeliDose of Monaco had a apparently lower MCS value than in Eclipse, indicating that a complex and small irradiation field was been used. However, CliDose and DeliDose demonstrated no significant difference. DeliDose plans exhibit no problem because CliDose is clinical acceptable plans.

### Limitation

This study has several limitations that should be considered. First, this study assessed dose distributions in two TPSs, but it remians unclear how to create clinically usable DeliDose plans in other TPSs. Additionally, this study trained and reproduced the dose distributions created in Monaco and completed it in Eclipse, which is a different TPS, but it is unclear currently whether the dose distributions created in Eclipse can be reproducted in Monaco. Reproducing the dose distribution created by Eclipse in Monaco may be difficult because Eclipse created a more concentrated dose distribution. Second, training data were calculated using Monaco. There were two potential biases in using the plan created in Monaco: one is that considerable variation of Monaco’s dose distribution could result in an inability to accurately learn the dose distribution for low to medium doses; the other is that the Monaco plan settings were not complex, which may have contributed to the poor dose reduction of the OAR. Third, the quality of DeliDose dose distribution depends on vendor-supplied optimization parameters. Currently, only one optimization parameter provied by vendor is available to reproduce DeliDose, future studies should evaluate the impact of this parameter. Forth, the results of this study were obtained on a small sample size test dataset (*n* = 10) with a single institution. Therefore, it may be necessary to use a multi-institution dataset or a larger amount of dataset to ensure that the results are generalizable.

## Conclusion

We created a deliverable whole pelvic VMAT plan using RatoGuide, which is a DL-based dose distribution prediction system, and evaluated its clinical validity.

We reproduced the dose distribution predicted by RatoGuide’s DD U-Net model on two TPSs to create deliverable plans (DeliDose), and both of them were well-behaved. DeliDose demonstrated inferior DVH metrics slightly compared to the human manually generated plan (CliDose), but radiation oncologist considered all DeliDose plans to be clinically acceptable and the GPR is also passed clinically. RatoGuide can significantly improve the efficiency of radiotherapy planning in clinical practice for whole pelvic VMAT irradiation of gynecologic cancers.

## Electronic supplementary material

Below is the link to the electronic supplementary material.

Supplementary Material 1

## Data Availability

it is difficult to public our dataset due to our hospital’s policy. Please contact Noriyuki Kadoya for data availability (E-mail: kadoya.n@rad.med.tohoku.ac.jp).
