# Supplementary material for: Evaluation of deliverable artificial intelligence-based automated volumetric arc radiation therapy planning for whole pelvic radiation in gynecologic cancer
Source: Sci Rep. 2025 Apr 30;15:15219. doi: 10.1038/s41598-025-99717-y (PMC12043927; doi:10.1038/s41598-025-99717-y)
Supplement: Supplementary file 1 — Supplementary Material 1 [file 41598_2025_99717_MOESM1_ESM.docx]

**Supplementary information**

**Evaluation of Deliverable Artificial Intelligence-Based Automated Volumetric Arc Radiation Therapy Planning for Whole Pelvic Radiation in Gynecologic Cancer**

Yushan Xiao, M.S.,^(1)^ Shohei Tanaka, Ph.D.,^(1)^ Noriyuki Kadoya*, Ph.D.,^(1)^ Kiyokazu Sato, M.S.,^(2)^ Yuto Kimura., Ph.D., ^(3)^ Rei Umezawa, M.D., Ph.D.,^(1)^ Yoshiyuki Katsuta, Ph.D.,^(1)^ Kazuhiro Arai, Ph.D.,^(1)^ Haruna Takahashi, M.S.,^(1)^ Hoshino Taichi, M.S.,^(1)^ Keiichi Jingu, M.D., Ph.D.,^(1)^

(1) Department of Radiation Oncology, Tohoku University Graduate School of Medicine, Sendai, Japan

(2) Radiation Technology, Tohoku University Hospital, Sendai, Japan

(3) Radiation Oncology Center, Ofuna Chuo Hospital, Kamakura, Japan


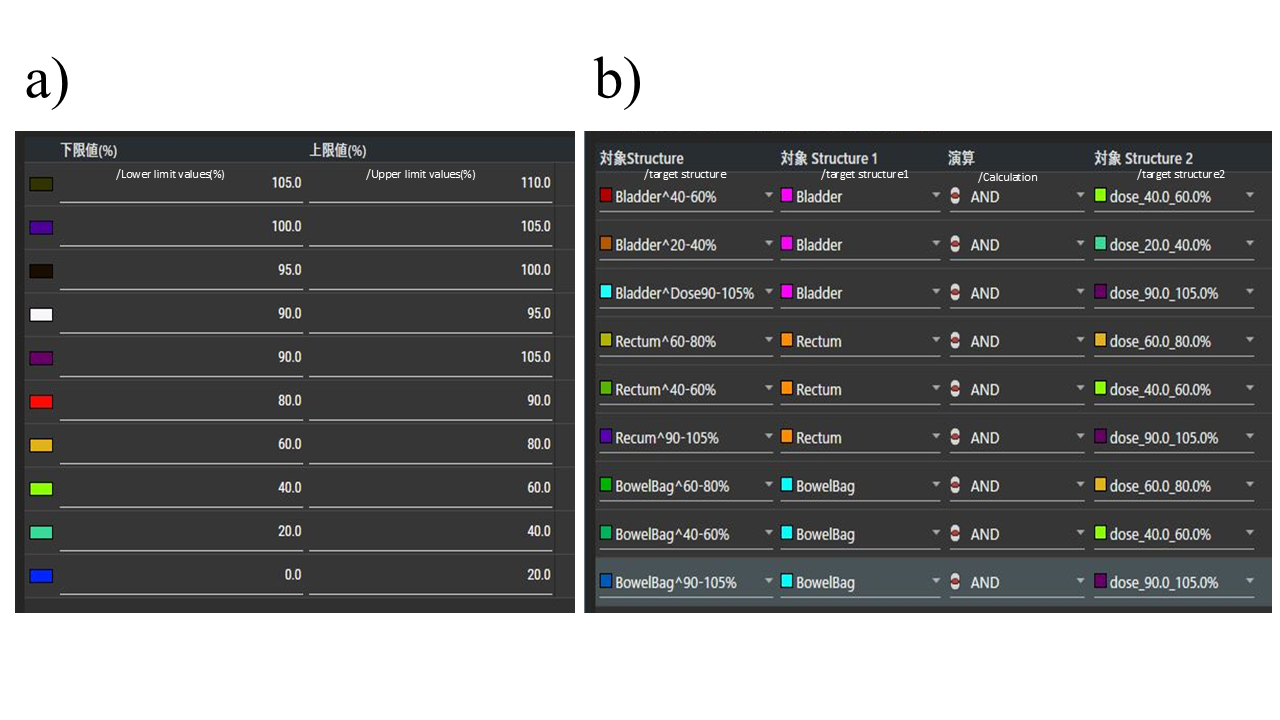


Supplementary Figure S1: **Template of** **the dose structure in increments of 5%–20% (a) and template of the overlapping region between the dose structure and OARs (b) in RatoGuide to create the dose structure.**


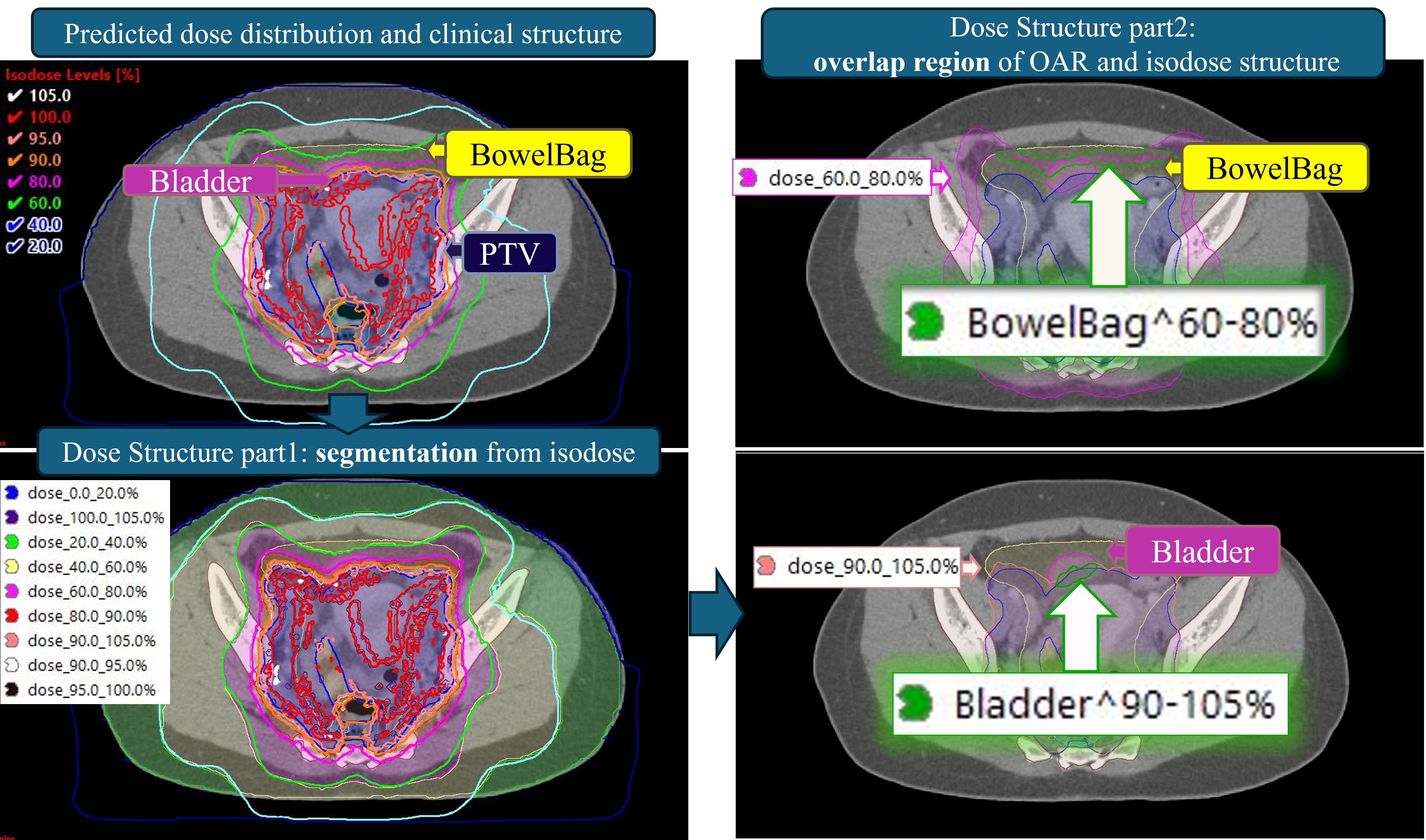


Supplementary Figure S2: **Workflow of the creation of a dose structure in RatoGuide.**


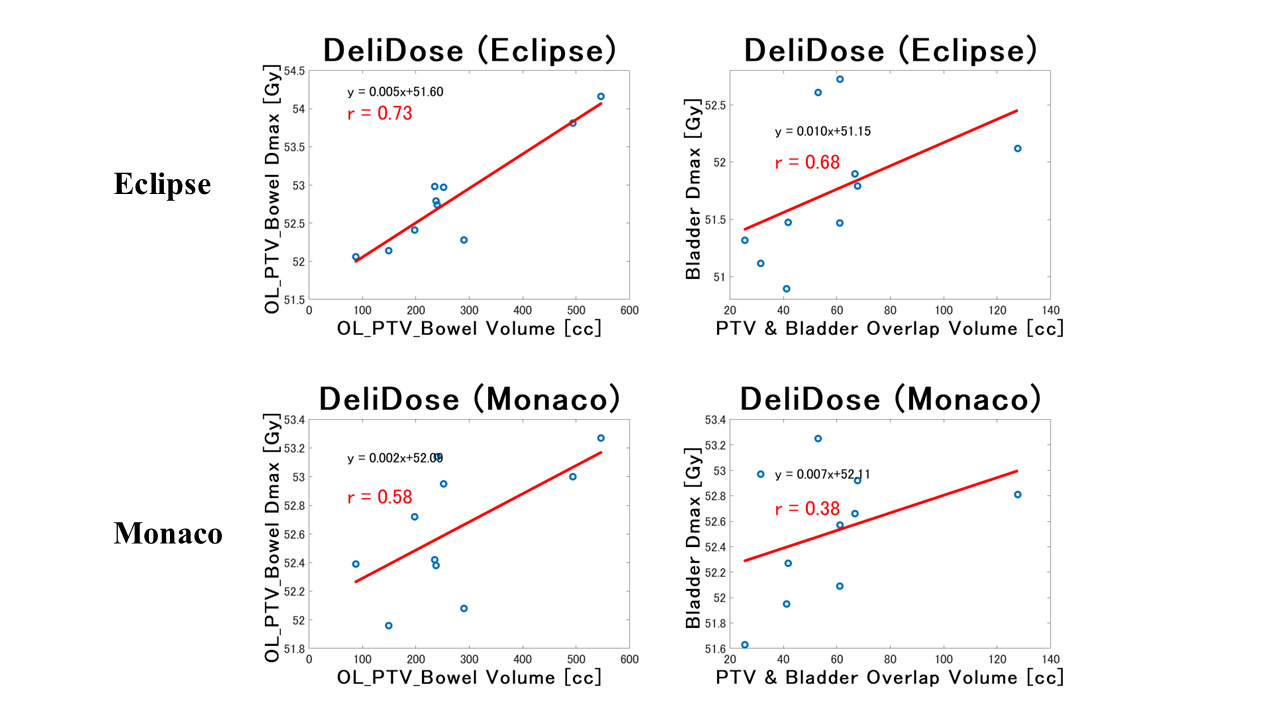


Supplementary Figure S3: **Correlation between the overlap volume between PTV and OARs and Dmax of OARs of DeliDose.**

Abbreviations:

DeliDose, deliverable dose; OL_PTV_Bowel, common region between the PTV and bowel bag.


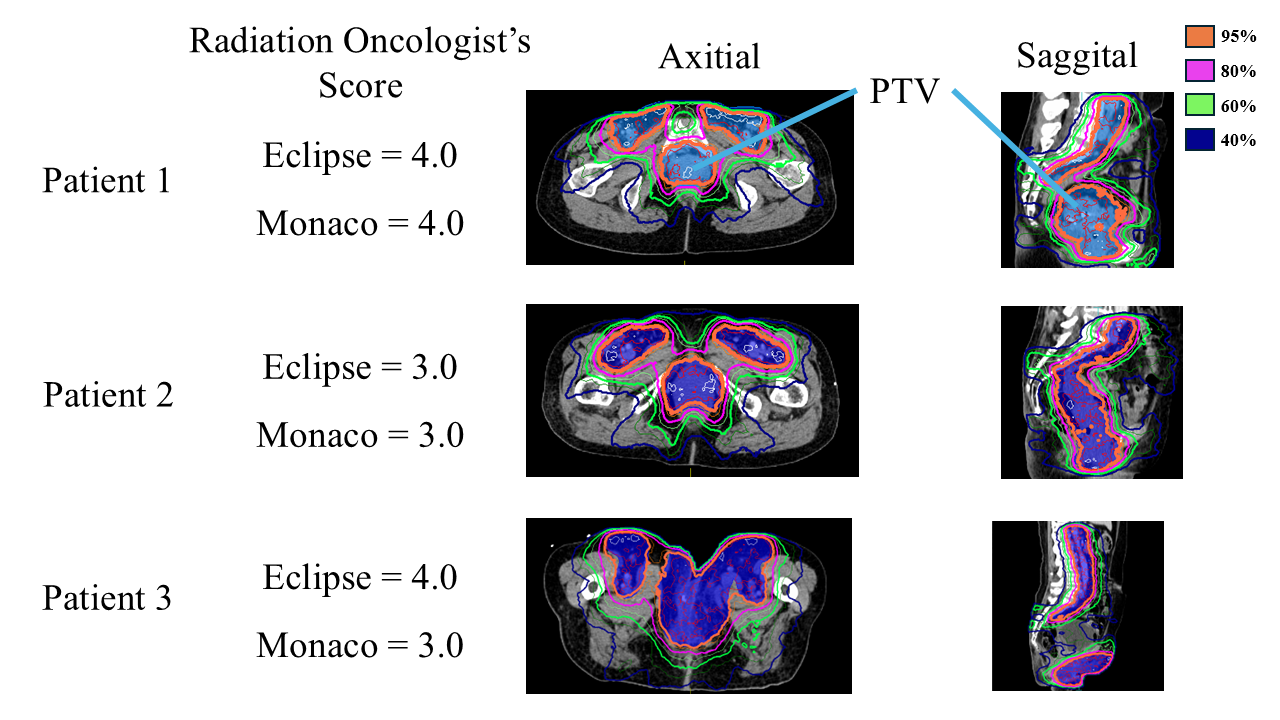


Supplementary Figure S4: **Radiation oncologist’s score for the three additional patients in whom planning is particularly difficult**. These three patients had targets including the inguinal lymph node region. Furthermore, Patient 3 had a very large PTV. Even in these patients in whom planning was difficult, the DeliDose plans achieved scores ≥ 3 (clinically acceptable) in both Monaco and Eclipse.


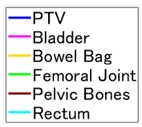

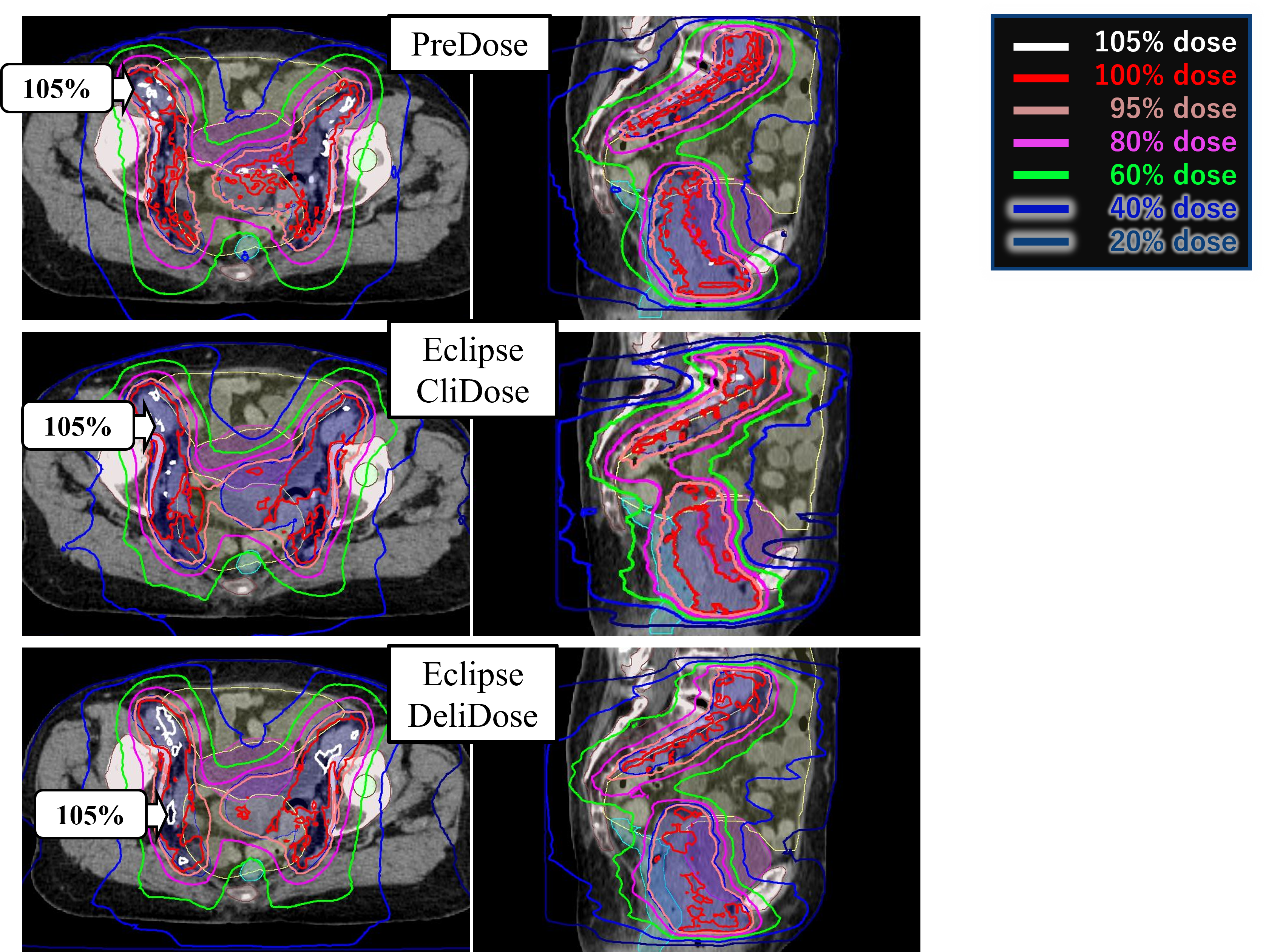


Supplementary Figure S5: **Dose distribution of PreDose, Clidose, and Delidose for the same patient.** White lines indicate hotspots

Abbreviations:

PreDose, predicted dose by deep learning model; DeliDose, deliverable dose; CliDose, clinical dose created manually

Supplementary Table S1: **Contour information of the training and test sets.**

| **Training set** | | Mean ± SD | Max | Min |
| --- | --- | --- | --- | --- |
| Volume of | PTV(cc) | 1330.5 ± 271.71 | 2323.73 | 823.56 |
|  | Bladder(cc) | 152.15 ± 97.97 | 599.46 | 26.21 |
|  | OL_PTV_Bowel(cc) | 306.20 ± 138.00 | 811.83 | 85.14 |
|  | OL_PTV_Bladder(cc) | 60.71 ± 30.57 | 206.52 | 22.68 |
| Length of PTV(cm) | | 21.87 ± 4.13 | 35.60 | 15.80 |
| **Test set** | | Mean ± SD | Max | Min |
| Volume of | PTV(cc) | 1310.12 ± 401.71 | 2454.74 | 958.37 |
|  | Bladder(cc) | 137.84 ± 59.87 | 269.93 | 48.94 |
|  | OL_PTV_Bowel(cc) | 273.01 ± 135.43 | 546.47 | 87.65 |
|  | OL_PTV_Bladder(cc) | 57.76 ± 27.16 | 127.72 | 25.58 |
| Length of PTV(cm) | | 21.38 ± 4.22 | 31.00 | 16.40 |

Abbreviations:

PTV: planning target volume; SD: standard deviation; OL_PTV_Bowel: overlap between PTV and bowel bag; OL_PTV_Bladder: overlap between PTV and bladder

Supplementary Table S2: **Dose constraints (JCOG 1402) in this study**

| Structure name | DVH metric | Optimal | Tolerable |
| --- | --- | --- | --- |
| Body | Dmax | <115% | <120% |
| PTV | D50% | =100% |  |
|  | D98% | >90% | >85% |
|  | D95% | >95% | >90% |
|  | D2% | <110% | <115% |
| OL_PTV_Bowel | Dmax | <105% | <110% |
| Rectum | V40 Gy | <85% | <95% |
|  | V50 Gy | <40% | <60% |
|  | Dmax | <110% | <120% |
| Bladder | V45 Gy | <50% | <70% |
|  | Dmax | <110% | <120% |
| Bowel Bag | V40 Gy | <40% | <50% |
| Pelvic Bones | V10 Gy | <85% | <95% |
|  | V40 Gy | <30% | <50% |
| Femoral Joint | V30 Gy | <40% | <60% |

Abbreviations:

Dmax: the maximum dose; Dx%: dose covering x% of the volume of the organ; OL_PTV_Bowel: overlap between PTV and bowel bag; VyGy: volume receiving yGy.

Supplementary Table S3: **Dose structure and description**

| Structure name | Description |
| --- | --- |
| preDose_100-105% | regions surrounded by 100-105% of the prescribed dose in the body |
| preDose_95-100% | regions surrounded by 95-100% of the prescribed dose in the body |
| preDose_90-95% | regions surrounded by 90-95% of the prescribed dose in the body |
| preDose_80-90% | regions surrounded by 80-90% of the prescribed dose in the body |
| preDose_60-80% | regions surrounded by 60-80% of the prescribed dose in the body |
| preDose_40-60% | regions surrounded by 40-60% of the prescribed dose in the body |
| preDose_20-40% | regions surrounded by 20-40% of the prescribed dose in the body |
| preDose_Bladder90-105% | regions surrounded by 90–105% of the prescribed dose within only the bladder |
| preDose_Bladder40-60% | regions surrounded by 40–60% of the prescribed dose within only the bladder |
| preDose_Bladder20-40% | regions surrounded by 20–40% of the prescribed dose within only the bladder |
| preDose_Rectum90-105% | regions surrounded by 90–105% of the prescribed dose within only the rectum |
| preDose_Rectum60-80% | regions surrounded by 60–80% of the prescribed dose within only the rectum |
| preDose_Rectum40-60% | regions surrounded by 40–60% of the prescribed dose within only the rectum |
| preDose_BowelBag90-105% | regions surrounded by 90–105% of the prescribed dose within only the bowel bag |
| preDose_BowelBag60-80% | regions surrounded by 60–80% of the prescribed dose within only the bowel bag |
| preDose_BowelBag40-60% | regions surrounded by 40–60% of the prescribed dose within only the bowel bag |
| preDose_PelvicBone0-20% | regions surrounded by 0–20% of the prescribed dose within only the pelvic bone |

Supplementary Table S4: **Eclipse optimization parameters for the DeliDose**

| Eclipse optimization parameters | | | | |
| --- | --- | --- | --- | --- |
| Structure name | constraint | Vol [%] | Dose [Gy] (50.4Gy = 100%) | Priority |
| preDose_100-105% | Upper | 0 | 50.9 Gy (101%) | 600 |
|  | Lower | 100 | 50.4 Gy (100%) | 500 |
|  | Lower | 100 | 49.9 Gy (99%) | 900 |
| preDose_95-100% | Lower | 100 | 49.9 Gy (99%) | 950 |
| PTV | Upper | 0 | 50.4 Gy (100%) | 600 |
|  | Upper | 0 | 51.91 Gy (103%) | 900 |
|  | Upper | 0 | 52.92 Gy (105%) | 1000 |
|  | Lower | 100 | 49.9 Gy (99%) | 950 |
| preDose_Bladder20-40% | Upper | 0 | 20.16 Gy (40%) | 700 |
| preDose_Bladder40-60% | Upper | 0 | 30.24 Gy (60%) | 700 |
| preDose_Bladder90-105% | Upper | 0 | 49.9 Gy (99%) | 800 |
| preDose_BowelBag40-60% | Upper | 0 | 27.72 Gy (55%) | 700 |
| preDose_BowelBag60-80% | Upper | 0 | 40.32 Gy (80%) | 700 |
| preDose_BowelBag90-105% | Upper | 0 | 49.9 Gy (99%) | 950 |
| preDose_20-40% | Upper | 0 | 20.16 Gy (40%) | 700 |
| preDose_40-60% | Upper | 0 | 30.24 Gy (60%) | 700 |
| preDose_60-80% | Upper | 0 | 40.32 Gy (80%) | 600 |
| preDose_80-90% | Upper | 0 | 45.36 Gy (90%) | 500 |
| preDose_90-95% | Upper | 0 | 47.88 Gy (95%) | 500 |
| preDose_PelvicBone0-20% | Upper | 0 | 7.56 Gy (15%) | 800 |
| preDose_Rectum40-60% | Upper | 0 | 30.24 Gy (60%) | 700 |
| preDose_Rectum60-80% | Upper | 0 | 40.32 Gy (80%) | 700 |
| preDose_Rectum90-105% | Upper | 0 | 49.9 Gy (99%) | 800 |

Abbreviations: Vol: volume; PTV: planning target volume.

Supplementary Table S5: **Monaco optimization parameters for DeliDose**

| Monaco optimization parameters | | | | | | | | |
| --- | --- | --- | --- | --- | --- | --- | --- | --- |
| Structure name | Cost Function | Manual | | Weight | Reference Dose [Gy]  (50.4Gy = 100%) | Shrink Margin [cm] | Iso Constraint  (50.4Gy = 100%) | Minimum Volume [%] |
| preDose_100-105% | Target Penalty | | ✓ | 2 |  |  | 50.904 (101%) | 95% |
| preDose_95-100% | Target Penalty | | ✓ | 2 |  |  | 48.384 (96%) | 99.50% |
| PTV | Target Penalty | | ✓ | 2 |  |  | 48.384 (96%) | 99.80% |
|  | Quadratic Overdose | | ✓ | 2 | 51.408 (102%) |  | 0.1 |  |
|  | Quadratic Overdose | | ✓ | 2 | 54.432 (108%) |  | 0.02 |  |
| preDose_Bladder40-60% | Quadratic Overdose | | ✓ | 1 | 30.24 (60%) | 0 | 0.04 |  |
| preDose_Bladder20-40% | Quadratic Overdose | | ✓ | 1 | 20.16 (40%) | 0 | 0.04 |  |
| preDose_Rectum60-80% | Quadratic Overdose | | ✓ | 1 | 40.32 (80%) | 0 | 0.04 |  |
| preDose_Rectum40-60% | Quadratic Overdose | | ✓ | 1 | 30.24 (60%) | 0 | 0.04 |  |
| preDose_BowelBag60-80% | Quadratic Overdose | | ✓ | 1 | 40.32 (80%) | 0 | 0.04 |  |
| preDose_BowelBag40-60% | Quadratic Overdose | | ✓ | 1 | 30.24 (60%) | 0 | 0.04 |  |
| preDose_PelvicBone0-20% | Quadratic Overdose | | ✓ | 1 | 9.072 (18%) | 0 | 0.02 |  |
| preDose_90-95% | Quadratic Overdose | | ✓ | 1 | 47.88 (95%) | 0.1 | 0.1 |  |
| preDose_80-90% | Quadratic Overdose | | ✓ | 1 | 45.36 (90%) | 0.2 | 0.1 |  |
| preDose_60-80% | Quadratic Overdose | | ✓ | 1 | 40.32 (80%) | 0.3 | 0.1 |  |
| preDose_40-60% | Quadratic Overdose | | ✓ | 1 | 30.24 (60%) | 0.6 | 0.1 |  |
| preDose_20-40% | Quadratic Overdose | | ✓ | 1 | 20.16 (40%) | 0.9 | 0.1 |  |
| preDose_BowelBag90-105% | Quadratic Overdose | | ✓ | 2 | 50.904 (101%) | Overall volume | 0.02 |  |
| preDose_Rectum90-105% | Quadratic Overdose | | ✓ | 1 | 50.4 (100%) | Overall volume | 0.1 |  |
| preDose_Bladder90-105% | Quadratic Overdose | | ✓ | 1 | 50.4 (100%) | Overall volume | 0.1 |  |

Abbreviations: PTV: planning target volume.

Supplementary Table S6: **Description of the scores according to radiation oncologist evaluation.**

| **Score** | **Scoring in radiation oncologist evaluation.** |
| --- | --- |
| 1 | Unacceptable irradiation of patients |
| 2 | Unacceptable irradiation to patients, but with a little improvement, irradiation is possible |
| 3 | Clinically irradicable level, but there are a few points that are of concern and could be improved. |
|  | (Patients can be irradiated) |
| 4 | Clinically irradicable level, but if only one or two points could be improved, it would be a perfect plan. |
| 5 | A perfect plan and there is absolutely nothing to be concerned about. |

Supplementary Table S7: **Dice coefficients for PreDose vs. DeliDose and CliDose vs. DeliDose for Monaco and Eclipse per 20% dose structure**

| Dose structure | Monaco | | Eclipse | |
| --- | --- | --- | --- | --- |
|  | PreDose vs. DeliDose | CliDose vs. DeliDose | PreDose vs. DeliDose | CliDose vs. DeliDose |
|  | Mean ± SD | Mean ± SD | Mean ± SD | Mean ± SD |
| 0-20% | 0.97 ± 0.007 | 0.97 ± 0.013 | 0.98 ± 0.007 | 0.97 ± 0.014 |
| 20-40% | 0.78 ± 0.029 | 0.72 ± 0.026 | 0.82 ± 0.016 | 0.75 ± 0.053 |
| 40-60% | 0.74 ± 0.022 | 0.68 ± 0.020 | 0.78 ± 0.013 | 0.69 ± 0.067 |
| 60-80% | 0.79 ± 0.014 | 0.70 ± 0.021 | 0.78 ± 0.016 | 0.69 ± 0.055 |
| 80-100% | 0.81 ± 0.018 | 0.79 ± 0.022 | 0.78 ± 0.028 | 0.79 ± 0.025 |
| 100-120% | 0.71 ± 0.025 | 0.71 ± 0.025 | 0.67 ± 0.032 | 0.75 ± 0.070 |

Abbreviations: SD: standard deviation.
